# Impact of qualitative endoscopic ultrasonography on fatty pancreas at a referral medical center

**DOI:** 10.1002/jgh3.12692

**Published:** 2021-12-12

**Authors:** Atsushi Kawamura, Kazuki Takakura, Yuichi Torisu, Yuji Kinoshita, Yoichi Tomita, Masanori Nakano, Takashi Yamauchi, Machi Suka, Kazuki Sumiyama, Shigeo Koido, Masayuki Saruta

**Affiliations:** ^1^ Division of Gastroenterology and Hepatology, Department of Internal Medicine The Jikei University School of Medicine Tokyo Japan; ^2^ UnMed Clinic Motomachi Kanagawa Japan; ^3^ Department of Public Health and Environmental Medicine The Jikei University School of Medicine Tokyo Japan; ^4^ Department of Endoscopy The Jikei University School of Medicine Tokyo Japan

**Keywords:** endoscopic ultrasonography, metabolic syndrome, nonalcoholic fatty liver disease, nonalcoholic fatty pancreas disease, pancreatic cancer

## Abstract

**Background:**

Taking advantage of the current advances in diagnostic imaging modalities, including endoscopic ultrasonography (EUS), and due to the increased attention to ectopic fat accumulation in the pancreas following the rising trend of metabolic syndrome, we qualitatively assessed the clinical implication of pancreatic steatosis by EUS in this study.

**Methods:**

The study included 243 patients that were divided into four groups. The correlation between the average echogenicity of the pancreas and that of the control organs and the key clinical data of all study patients were collectively analyzed. The cut‐off point of the pancreas‐control (PC) ratio in EUS and liver‐control (LC) ratio on abdominal ultrasound were determined from the population distribution and the obtained median values.

**Results:**

With the cut‐off point of 1.30 for the PC ratio and 1.20 for the LC ratio, sex, the Brinkman index, habitual alcohol drinkers, and fatty pancreas were significant factors. The associations between each relevant factor in fatty pancreas, metabolic syndrome in the fatty liver group, and age in the pancreatic cancer group were all significant in the analysis. In addition, we investigated whether the PC ratio differed according to age and staging in pancreatic cancer patients. Interestingly, the PC ratio was lower in the advanced stage group than in the early‐stage group.

**Conclusion:**

Our results suggest that, irrespective of the degree, ectopic fat infiltration in the pancreas could be a specific clinical phenotype of serious pancreatic diseases, including pancreatic cancer, especially in high‐risk patients.

## Graphical abstract

In this study, the degree of fat infiltration in the pancreas was first assessed using EUS, which is the most reliable imaging modality. In the cases of pancreatic cancer and pancreatitis, almost all had a value above the standard value for fatty metamorphosis in the pancreas. This suggests that pancreatic cancer and pancreatitis are strongly associated with fatty conditions in the pancreas.

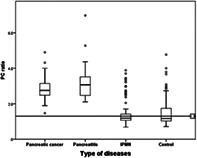

## Introduction

The limited visualization of the whole pancreas using abdominal ultrasonography (US) made early detection of pancreatic cancer difficult. However, with the current advances in imaging modalities, including endoscopic ultrasonography (EUS), the visualization of the whole pancreas has dramatically improved, especially in obese patients.^1^, ^2^, ^3^ Ectopic fat deposition in the pancreas has received increased attention over the last few years, owing to the rising prevalence of metabolic syndrome worldwide. Indeed, several studies regarding pancreatic steatosis have been reported, and similar to nonalcoholic fatty liver disease (NAFLD), the condition has been defined as nonalcoholic fatty pancreas disease for ectopic fat infiltration of the pancreas in the absence of excess alcohol intake.^4^, ^5^, ^6^, ^7^

Interestingly, the clinical relationship between these lipid metabolism disorders in the liver and pancreas has been determined,^8^, ^9^, ^10^ and NAFLD is the hepatic manifestation of metabolic syndrome.^11^, ^12^ Furthermore, previous studies have shown that fat deposition in the pancreas is a possible risk factor for pancreatic cancer.^13^, ^14^ In addition, Kashiwagi *et al*. reported that intraductal papillary mucinous neoplasm (IPMN), a premalignant lesion of pancreatic cancer, may develop secondary to pancreatic steatosis, which could be an overlapping risk factor for pancreatic cancer and IPMN.^15^ Moreover, Taylor *et al*. clearly demonstrated that type‐2 diabetes (DM), a major pancreatic disease, is triggered by an overspill of fat from the liver into the pancreas based on their twin cycle hypothesis.^16^, ^17^ Although dyslipidemia should be more noteworthy and elucidated based on the recent trends, the degree of fat accumulation in the pancreas and its correlation with underlying diseases or the risk of pancreatic diseases have not been assessed quantitatively because of the limitation in the resolution of diagnostic images.

In this study, we evaluated the clinical implication of the degree of pancreatic steatosis in patients with pancreatic diseases by using EUS in addition to analyzing its relationship with the underlying conditions and the pathogenic risks of pancreatic diseases.

## Methods and analysis

### Patient characteristics

**Figure 1:**
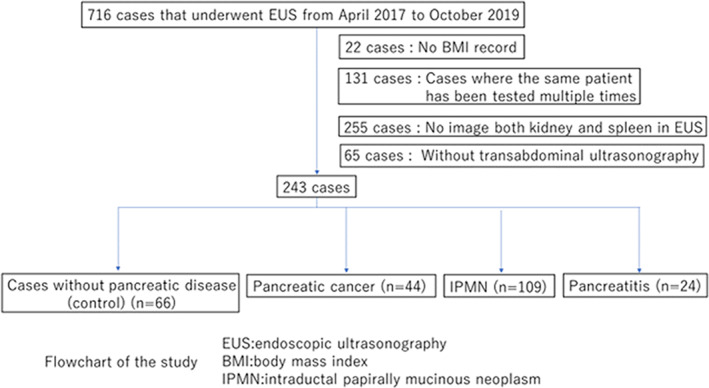
Flow chart of the study. A total of 716 patients who underwent EUS at the Jikei University Kashiwa Hospital from April 2017 to October 2019 were eligible for the study. Among these patients, we excluded 22 patients due to the lack of BMI records, 131 patients because of multiple examinations, 255 patients due to the lack of control images, and 65 patients due to the lack of liver images. Finally, 243 patients were included in the final analysis and were divided into four groups: control group, pancreatic cancer group, IPMN group, and pancreatitis group.

**Figure 2:**
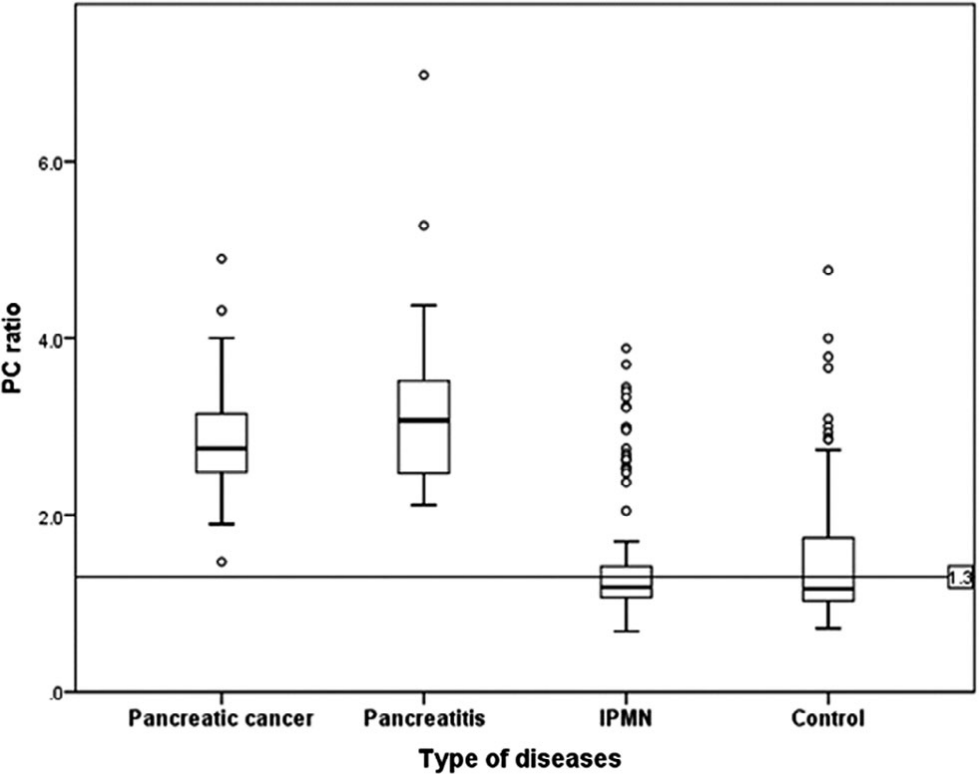
Box plot for each disease group. The PC ratios of the pancreatic cancer and pancreatitis groups on EUS were far above the cut‐off point of 1.3, compared with the other two groups. Therefore, we evaluated subsequent analyses without these two diseases.

In this retrospective observational study, 716 consecutive patients who underwent EUS examinations between April 2017 and October 2019 at The Jikei University Kashiwa Hospital were eligible for inclusion in the analysis. As shown in the scheme (Fig. 1), the exclusion criteria were the lack of necessary data, multiple tests in one patient, and the lack of EUS images and/or liver abdominal US images for comparison. A total of 243 patients were divided into four groups: patients without any pancreatic diseases (*n* = 66), patients with pancreatic cancer (*n* = 44), patients with IPMN (*n* = 109), and patients with pancreatitis (*n* = 26) (Fig. 2). Clinical data, such as age, sex, body mass index (BMI), and laboratory data including low‐density and high‐density lipoprotein, triglycerides, amylase, and lipase of all the study patients were collected and summarized in our database designed for this work. In addition, medical histories of DM, hypertension (HT), hyperlipidemia (HLP), and history of smoking and drinking were similarly recorded based on self‐reported information. A summary of the data collected from the study patients is presented in Table 1. The study protocol was approved by the ethics committee of The Jikei University School of Medicine, Tokyo, Japan (authorization number: 31–179). The review board approved and waived the need for written informed consent from the participants due to the retrospective, noninterventional nature of this study. We have read the Declaration of Helsinki and have followed the recommended guidelines in this study.

**Table 1:** Baseline patient characteristics (*n* = 243)

		Pancreatic cancer (*n* = 44)	Pancreatitis (*n* = 24)	IPMN (*n* = 109)	Negative control (*n* = 66)	*P* value
Age, mean (±SD), years	66.45 ± 12.12	66.57 ± 11.43	65.16 ± 11.84	68.40 ± 10.39	63.61 ± 14.72	0.08
Sex, male (%)	133 (54.7)	24 (54.5)	22 (91.7)	50 (45.9)	37 (56.1)	0.001
Brinkman Index 0 (%)	129 (53.1)	19 (43.2)	3 (12.5)	73 (67.0)	34 (51.5)	<0.001
1–599	61 (25.1)	9 (20.5)	11 (45.8)	23 (21.1)	18 (27.3)	
600≦	53 (21.8)	16 (36.4)	10 (41.7)	13 (11.9)	14 (21.2)	
Alcohol addict (6–7 day/week) (%)	42 (17.3)	12 (27.3)	7 (29.2)	13 (11.9)	10 (15.2)	0.049
BMI 25≦	48 (19.8)	6 (13.6)	5 (20.8)	22 (20.2)	15 (22.7)	0.697
Metabolic syndrome (+) (%)	25 (10.3)	5 (11.4)	3 (12.5)	10 (9.2)	7 (10.6)	0.953
Fatty liver (LC ratio > 1.2) (%)	88 (36.2)	13 (29.5)	7 (29.2)	39 (35.8)	29 (43.9)	0.38
Fatty pancreas (PC ratio > 1.3) (%)	126 (51.9)	44 (100)	24 (100)	35 (32.1)	23 (34.8)	<0.001

### Assessment of pancreatic steatosis by EUS‐based measurement

EUS (GF‐UCT260; Olympus, Tokyo, Japan) with ultrasound observation systems (EU‐ME2; Olympus, Tokyo, Japan) were performed under sedation using a linear array echoendoscope in a standard fashion. We analyzed the correlation between the degree of pancreatic steatosis based on the average EUS values and clinical characteristics of all study patients.

### Qualitative analysis of average echogenicity from whole pancreas by EUS

**Figure 3:**
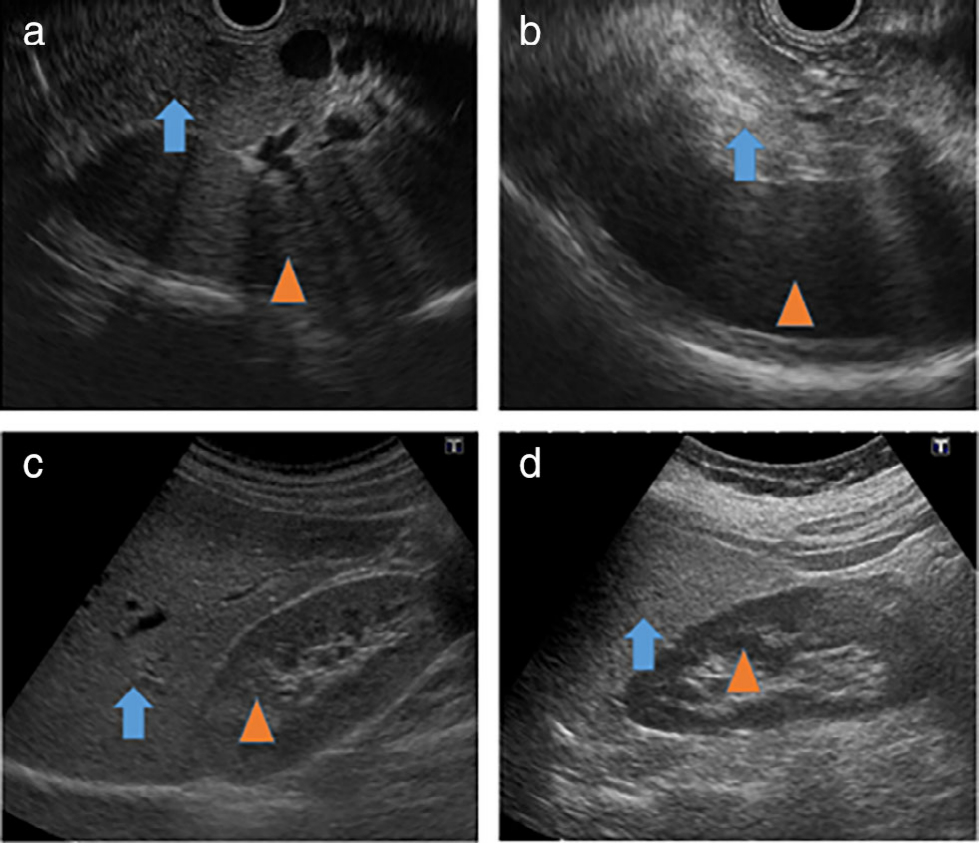
Representative images. (a) Iso‐dense image of the normal pancreas (Arrow) with the spleen (Arrowhead). (b) High‐density image of fatty pancreas (Arrow) with the spleen (Arrowhead). (c) Iso‐dense image of the normal liver (Arrow) with the right kidney (Arrowhead). (d) High‐density image of fatty liver (Arrow) with the right kidney (Arrowhead).

To prevent biased evaluation due to the uneven distribution of ectopic fat deposition in the pancreas, we set regions of interest (ROIs) at three different areas in the pancreas and calculated the average echogenicity of the ROIs for all study patients. The average echogenicity of the pancreas was compared with that of the spleen or left kidney as the control, and the ratio of the echogenic value of the patient was calculated by dividing that of the pancreas by that of the control. Similarly, the degree of liver steatosis was measured by setting ROIs at three different areas in the liver during abdominal US examinations and calculating the average echogenicity by comparing it with the echogenicity of the right kidney. From the population distribution and the obtained median value, the cut‐off points of the pancreas‐control (PC) ratio in EUS and liver‐control (LC) ratio in abdominal US were determined to be 1.3 and 1.2, respectively. The average value obtained by the three observers was taken as the measured value. The representative images of high echogenicity of the fatty pancreas and liver versus those of the normal ones are shown in Figure 3.

### Metabolic syndrome and smoking index

Considering our targeted study population in Japan, we applied the Japanese criteria for metabolic syndrome.^18^ According to the criteria, metabolic syndrome is diagnosed if the patient has central adiposity plus two or more of the following: 1) Increased concentration of triglycerides ≥150 mg/dL or reduced concentration of HDL cholesterol <40 mg/dL; 2) elevated blood pressure as systolic blood pressure ≥ 130 mmHg or diastolic blood pressure ≥ 85 mmHg, or treatment of previously diagnosed hypertension; and 3) elevated fasting plasma glucose concentration ≥ 110 mg/dl. The thresholds for waist circumference to define central adiposity were ≥85 cm for men and ≥90 cm for women. The severity level of smoking was assessed using the Brinkman index. The Brinkman index value was obtained by multiplying the average number of cigarettes smoked per day by the number of years the person has smoked.

### Statistical analysis

All analyses were conducted using SPSS version 25 (IBM Corp., Armonk, NY, USA). We set the cut‐off point for the PC ratio for the degree of fatty pancreas following the population distribution of the PC ratio and the obtained median value (Fig. 2).

**Figure 4:**
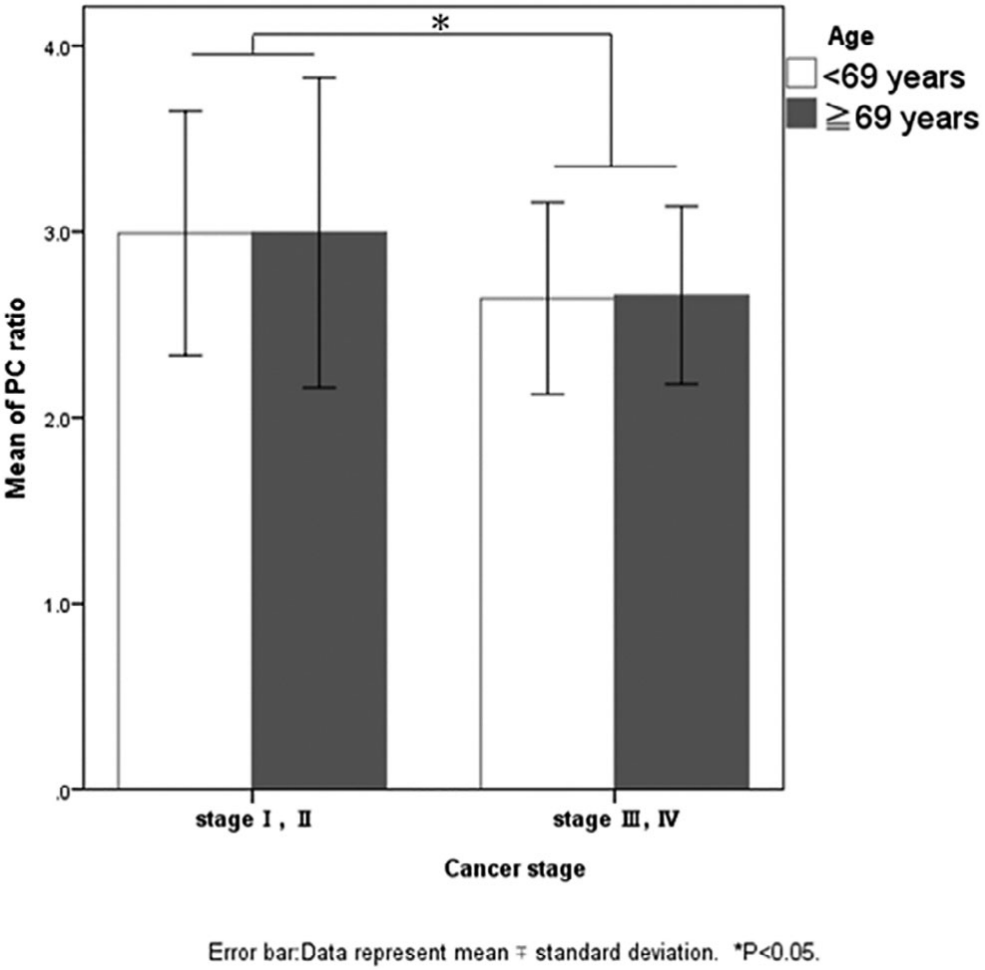
Relationship between fatty pancreas and pancreatic cancer staging. Two‐way analysis of variance was used to identify the pancreatic cancer groups based on the population age (median divided into two groups) and cancer stage (early stage and advanced stage groups). No age‐stage interaction was observed, but the PC ratio on EUS was lower in the advanced stage group than in the early‐stage group (*P* = 0.035).

First, for each variable, cross‐tabulation and Chi‐square tests were conducted based on the type of disease (Table 1). Since almost all of the patients with pancreatic cancer and pancreatitis were confirmed to have fatty pancreas, we performed Chi‐square tests and multiple logistic regression analyses to examine which factors were associated with fatty pancreas among the IPMN and control groups (Table 2). To examine the association between the relevant factors and PC ratio in patients with pancreatic cancer and those with fatty liver in the IPMN and control groups, we conducted multiple regression analyses using the PC ratio as the dependent variable (Table 3). We did not perform multiple regression analyses for those with pancreatitis because the sample size of this group was small. Finally, to examine whether the PC ratio differed by age and cancer stage among those with pancreatic cancer, we conducted a two‐way analysis of variance (Fig. 4). We divided the participants' ages into two categories by median (i.e., 69 years) and cancer stage into two categories: early stage (stage I or II) and advanced stage (stage III or IV). Statistical significance was set at *P* < 0.05.

**Table 2:** Fatty pancreas characteristics of study group (IPMN, negative control)

	Nonfatty pancreas (*n* = 117)	(%)	Fatty pancreas (*n* = 58)	(%)	*P* value
Age	65.93 ± 12.52		67.93 ± 12.10		0.256
Sex					0.001
Male	48	41.0	39	67.2	
Female	69	59.0	19	32.8	
Smoking Index					0.041
0	79	67.5	28	483	
1–599	24	20.5	17	29.3	
600≦	14	12.0	13	22.4	
Alcohol intake	13	11.1	10	17.2	0.259
Bill 25≦	10	8.5	27	46.6	<0.001
Metabolic syndrome	4	3.4	13	22.4	<0.001
Fatty liver	18	15.4	50	86.2	<0.001
IPMN	74	63.2	35	60.3	0.737
Control	43	36.8	23	39.7	0.737

**Table 3:** Multiple regression analysis of factors related to fatty pancreas among factors with high PC ratio

	Fatty liver (*n* = 68)		Pancreatic cancer (*n* = 44)	
	β	*P* value	β	*P* value
Age	–0.125	0.307	–0.34	0.041
Sex	0.094	0.476	0.24	0.221
Brinkman Index	0.119	0.346	0.047	0.806
Alcohol addict (6–7 day/week)	0.029	0.818	0.224	0.135
Metabolic syndrome (+)	0.32	0.011	0.262	0.77

## Results

### Patient characteristics

A total of 243 patients were included in the analysis. Figure 2 depicts the distribution of the PC ratio in EUS according to the type of disease. Based on the population distribution, we set the cut‐off point at 1.3 for the PC ratio for fatty pancreas in the present study. The clinical characteristics of the patients are presented in Table 1. The mean age was 66.5 ± 12.1 years (range, 20–87); 133 patients were male (54.7%) and 110 were female (45.3%). There were 48 (19.8%) patients with a BMI ≤25, 25 (10.3%) had metabolic syndrome, 88 (36.2%) had fatty liver, and 126 (51.9%) had fatty pancreas. Dividing the degree of smoking with the Brinkman index, the value of 129 patients (53.1%) were 0, no smoking, 61 (25.1%) were 1–599, moderate smoking, and 53 (21.8%) were ≤ 600, heavy smoking. The number of habitual alcohol drinkers, defined as those drinking alcohol over 6 days in a week, was 42 (17.3%). The number of patients with pancreatic cancer, chronic pancreatitis, and no specific diseases as the control in the study were 44, 24, and 66, respectively. Sex (*P* = 0.001), the Brinkman index value (<0.001), habitual alcohol drinker (0.049), and fatty pancreas (0.001) showed significant results in the Chi‐square test (Table 1).

### Comparison of patients with and without hyperechogenic pancreas

First, since all the patients with pancreatic cancer and pancreatitis were confirmed to have fatty pancreas (PC ratio > 1.3), we performed Chi‐square tests and multiple logistic regression analyses to evaluate which factors were associated with fatty pancreas among the IPMN and control groups (Table 2). Among the 175 patients in these groups, the factors related to fatty pancreas were compared. Sex (0.001), the Brinkman index (0.041), BMI ≤25 (<0.001), metabolic syndrome (<0.001), and fatty liver (<0.001) were found to be significant factors (Table 2).

Second, univariate and multivariate analyses were performed to investigate the causes of pancreatic steatosis only in the IPMN and control groups (*n* = 175). The multivariate analysis revealed that the factors independently related to fatty pancreas were sex (OR: 5.1, 95% CI: 1.6–15.6), metabolic syndrome (OR 10.3, 95% CI: 1.9–54.5), and fatty liver (OR 42.4, 95% CI: 14.6–122.3). The correlation coefficient between the PC ratio in EUS and the LC ratio in abdominal US was as high as 0.84.

We also analyzed the additional effects of smoking and drinking, but these were not significant (data not shown).

### Comparison of each factor in fatty pancreas

Next, to examine the association between the relevant factors and the PC ratio among patients with pancreatic cancer and those with fatty liver in the IPMN and control groups, we conducted multiple regression analyses using the PC ratio as the dependent variable (Table 3). The only factors that showed significance were metabolic syndrome in the fatty liver group (*P* = 0.011) and age in the pancreatic cancer group (*P* = 0.041) (Table 3). This suggests that smoking and drinking did not directly affect the results. Additionally, we analyzed the combined effect of some factors; however, none of the combinations showed a significant difference (data not shown).

### The relationship between fatty pancreas and pancreatic cancer staging

Finally, to investigate whether the PC ratio differs based on the patient's age and cancer stage among the pancreatic cancer group, we conducted a two‐way analysis of variance (Fig. 4). No interaction between the stage and age was observed, but the PC ratio was lower in the advanced stage group than in the early‐stage group (*P* = 0.035) (Fig. 4). This suggests that the degree of fatty infiltration in the pancreas decreases as cancer progresses.

## Discussion

This study conducted in patients who underwent EUS in our hospital has demonstrated that ectopic fat accumulation would be a specific clinical phenotype in some major pancreatic diseases, including pancreatic cancer, regardless of its degree. In this study, the degree of fat infiltration in the pancreas was first assessed using EUS, which is the most reliable imaging modality. In the cases of pancreatic cancer and pancreatitis, almost all had a value above the standard value for fatty metamorphosis in the pancreas (Fig. 2, Table 1) and our data were consistent with previous works which showed that prevalence of fatty pancreas is high in patients with pancreatic cancer and pancreatitis.^19^, ^20^ This suggests that pancreatic cancer and pancreatitis are strongly associated with fatty conditions in the pancreas. Except for the patients with pancreatic cancer and pancreatitis, the assessment showed that the male gender, the metabolic syndrome, and being in the fatty liver group were significant factors in the development of a fatty pancreas (Tables 2).

Our results were consistent with those of previous reports,^12^ and supports the fact that visceral fat accumulates more easily in men than in women.^21^ Moreover, because a strong relationship between fatty pancreas and metabolic syndrome was revealed in the study (OR 10.3), fatty pancreas could be an initial imaging sign for a major disease. Additionally, our data demonstrated the relationship between the PC ratio in EUS and the LC ratio in abdominal US. This means that fat accumulation in the pancreas can occur via the same mechanism of visceral fat accumulation.

Curiously, metabolic syndrome, DM and HT, and HT alone showed significant differences in the univariate and multivariate analyses, respectively (data not shown). Further research is necessary in the future.^22^, ^23^

In addition, although not severe, our results showed a marked tendency for fatty pancreas, age, and metabolic syndrome to exacerbate the condition of patients with pancreatic cancer and fatty liver. This means that older patients with metabolic syndrome have the highest risk of developing pancreatic cancer; therefore, they are potential candidates for an annual screening test by EUS. Additionally, our data demonstrated that patients with advanced pancreatic cancer have a lower level of fatty accumulation in the pancreas compared with early‐stage pancreatic cancer patients. Fatty conditions are alleviated based on the deterioration of nutritional status, and pro‐inflammatory factors such as focal cytokines were more infiltrated. Moreover, the body weights of patients with advanced pancreatic cancer decreased as the disease progressed; thus, no patients will have met the criteria for metabolic syndrome. We believe this is the reason why metabolic syndrome was not a significant factor in our analysis.

There are several limitations in this study. First, this study lacks histological evidence of pancreatic steatosis, and some pancreatic cancer cases were clinically diagnosed without pathological evidence. Although pancreatic tissue samples were obtained by EUS‐guided fine‐needle aspiration in several study patients, the tissues were obtained from the involved areas and did not represent an overall picture of the condition of the pancreas. Therefore, we could not refer to any histological data in this study. Second, this study lacks a healthy control population. Intrinsically, EUS, which is a relatively invasive procedure, should not be performed in healthy persons without any valid indication. This results in a *loss* of *generalizability in our findings*. The third limitation is that the study did not consider the fibrotic changes when evaluating the echogenicity of the pancreas. Similar to the liver, we agree that echogenicity can decrease in the pancreas depending on the progression of the fibrotic changes.^24^, ^25^ On the other hand, in some chronic pancreatitis cases it may be possible that the coarse pancreatic parenchyma, which reflects fibrosis or acinar cell depletion rather than fat deposition, were evaluated as hyperechoic pancreas. Recently, the advancements in diagnostic imaging have allowed the assessment of not only the severity of steatosis but also the extent of fibrosis in the liver.^26^, ^27^, ^28^ As a result, the improvement in the qualitative diagnosis for steatosis and fibrosis in the pancreas is greatly anticipated.

To our knowledge, this is the first study that utilized EUS measurements that focused on fatty pancreas for pancreatic cancer staging. We agree that it is difficult to use EUS as a screening modality because of its high cost and the special skill. However, considering the visual limitation on the pancreas when using abdominal US especially in obese patients, it would be easy to overlook the curable and early stage of pancreatic cancer. Patients with severe fatty infiltration in the pancreas have a higher risk of pancreatic cancer; thus, detailed observation by EUS measurement is essential for early detection.

Compared with previous studies, our data were derived from qualitative assessments of the degree of fatty pancreas, and it would provide objective evidence without any difficulties in judging the appearance of the pancreatic parenchyma and pancreatic ducts.^3^

Taken together, there should be more focus on fatty pancreas, and the background diseases should also be examined carefully to prevent the development of malignancy, especially in patients with metabolic syndrome and fatty liver disease. However, our study included pancreatic cancer and pancreatitis who had a stronger tendency to develop fatty pancreas, which would indicate bias. Further investigations to confirm our findings are required in the future.

## Conclusion

We objectively assessed the clinical implications of the degree of pancreatic steatosis in patients with pancreatic diseases utilizing EUS measurements. In addition, we analyzed the relationship of the degree of pancreatic steatosis with the underlying conditions and the pathogenic risks of pancreatic diseases, mainly pancreatic cancer. Our findings indicate that ectopic fat accumulation in the pancreas could be a specific clinical phenotype of major pancreatic diseases, especially in high‐risk patients, regardless of its degree.

## Ethics approval statement

All procedures performed in studies involving human participants were in accordance with the ethical standards of the institutional research committee and with the 1964 Helsinki declaration and its later amendments or comparable ethical standards.

## Patient consent statement

This retrospective study was approved by the institutional review board, and the requirement to obtain informed consent was waived.

## Data Availability

The data that support the findings of this study are available from the corresponding author upon reasonable request.
